# Variability in the amount of homoeologous pairing among F1 hybrids

**DOI:** 10.1093/aobpla/plw030

**Published:** 2016-06-02

**Authors:** Lidia Poggio, Eduardo Greizerstein, María Ferrari

**Affiliations:** ^1^CONICET (Argentina); ^2^Cátedra de Mejoramiento Genético, Facultad de Ciencias Agrarias, Universidad Nacional de Lomas de Zamora (UNLZ), Provincia Buenos Aires, Argentina; ^3^Facultad de Ciencias Veterinarias, Universidad de Buenos Aires, Ciudad Autónoma de Buenos Aires, Argentina

**Keywords:** Glandularia*;* homoeologous pairing, pairing regulator genes, variability in the hybrid meiotic behaviour

## Abstract

Genes involved in the exclusive pairing of homologous chromosomes have been described in several polyploid species but little is known about the activity of these genes in diploids (which have only one dose of each homoeologous genome). Analysis of the meiotic behaviour of species, natural and artificial hybrids and polyploids of *Glandularia* suggests that, in allopolyploids where homoeologous genomes are in two doses, regulator genes prevent homoeologous pairing. The different meiotic phenotypes in diploid F1 hybrids between *Glandularia pulchella* and *Glandularia incisa* strongly suggest that these pairing regulator genes possess an incomplete penetrance when homoeologous genomes are in only one dose. Moreover, the meiotic analysis of natural and artificial F1 hybrids suggests that the genetic constitution of parental species influences the activity of pairing regulator genes and is mainly responsible for variability in the amount of homoeologous pairing observed in diploid hybrids. In *Glandularia*, the pairing regulator genes originated in South American diploid species. The cytogenetic characteristics of this genus make it a good model to analyse and explore in greater depth the activity of pairing regulator genes at different ploidy levels.

## Introduction

Correct segregation of chromosomes during meiosis is vital for the success of polyploid species which contain more than two sets of chromosomes that need to be sorted out during meiosis to produce balanced gametes. Genetic determination of exclusive paring between homologous chromosomes or cytological diploidization is the process by which meiosis in polyploids leads to chromosomally and genetically balanced gametes and has been described in several species of allopolyploid origin (Bhullar *et al.* 2014; Cifuentes *et al.* 2010; Jenczewski *et al.* 2003; Jenczewski and Alix 2004). Little is known about the activity of genes contributing to the cytological diploidization of polyploids. The best understood is the Ph1 locus of wheat, which suppresses pairing between homoeologous genomes. This gene may have multiple functions during meiosis and all the evidence suggest that Ph1 may behave like a master co-ordinator locus that controls the transcription of meiotic genes. Different authors have reported that Ph1 is involved in the onset of meiosis, has an effect on premeiotic chromosome arrangement, synchronizes chromatin remodelling and contributes to the clustering of the telomeres as a bouquet facilitating homologue recognition. Also, this locus could contribute to the fidelity of synapsis and crossover formation (Cifuentes *et al.* 2010; Colas *et al.* 2008; Feldman and Levy 2012; Holm and Wang 1988; Mikhailova *et al.* 1998; Zhou and Pawlowski 2014).

There are few studies about the activity of pairing regulator genes in diploids where there is only one set of each homoeologous genome. *Glandularia* is a useful model genus in which to analyse mechanisms involved in homologous/homoeologous chromosome pairing at different ploidy levels through the study of meiotic behaviour in species, hybrids and polyploids.

Species of *Glandularia* have a North-South American disjoint distribution (O’Leary and Peralta 2007; Peralta and Múlgura 2011). Many species, hybrids and polyploids hold great ornamental potential (Gonzalez Roca *et al.* 2015; Imhof *et al.* 2013). South American species are largely diploid (2*n* = 2*x* = 10) while those from North American are exclusively hexaploid (2*n* = 6*x* = 30) or tetraploid (2*n* = 4*x* = 20) (Poggio *et al.* 1993; Schnack and Covas 1945a,b; Schnack and González 1945; Schnack and Solbrig 1953; Schnack *et al.* 1959; Turner and Powell 2005; Umber 1979). *Glandularia* species typically showed normal pairing and segregation of chromosomes at metaphase I irrespective of the level of ploidy (Arora 1978; Khoshoo and Arora 1969; Poggio *et al.* 1993; Solbrig *et al.* 1968).

Schnack and Fehleisen (1955) reported that in the artificial diploid hybrid *Glandularia* *laciniata* × *Glandularia* *peruviana* 86% of its chromosomes form bivalents but only 12% of the chromosomes showed quadrivalents in the artificial allotetraploid. In *G. peruviana* × *Glandularia* *megapotamica* another diploid hybrid, they found all the chromosomes formed bivalents, and only 50% formed quadrivalents in the artificial allopolyploid. These authors postulated that the formation of multivalents in the polyploid was related to the degree of homology of parental species.

Solbrig *et al.* (1968) made artificial crosses between the North American hexaploid species *G. elegans* (2*n* = 30 = 6*x*, A^2^A^2^CCDD), and two diploid South American, *G. peruviana* (2*n* = 10, BB) and *G. pulchella* (2*n* = 10, A^1^A^1^). They found that the artificial F1 hybrids *G. pulchella* × *G. peruviana* (2*n* = 10, A^1^B) presented five bivalents indicating homoeologous pairing between the A^1^ and B genomes, while the artificial allotetraploid (2*n* = 20, A^1^A^1^BB) formed ten bivalents indicating the suppression of homoeologous pairing between A^1^ and B. On other hand, Solbrig *et al.* (1968) observed that intergenomal pairing between homoeologous chromosomes was suppressed in *G. elegans*. The F1 hybrids *G. elegans* × *G. peruviana* (2*n* = 20, A^2^BCD) had five tetravalents due to homoeologous pairing between A^2^BC and D, but the allotetraploid A^2^A^2^BBCCDD had 20 bivalents at metaphase I. Based on this information, Solbrig *et al.* (1968) proposed that *G. elegans* was an allopolyploid that showed complete diploid-like meiotic behaviour. Similar results were obtained by Khoshoo and Arora (1969) when they analyzed the meiotic behaviour of some species of *Verbena*, which is, in evolutionary terms, very close to *Glandularia.* These authors studied an artificial F_1_ hybrid of a North American hexaploid and a South American diploid and found that the hexaploid species was an allopolyploid involving three homoeologous genomes that showed complete diploid-like meiotic behaviour. These authors suggested that hexaploid species of *Verbena* possesses a multivalent suppressor system. All these observations identify homoelogous chromosome pairing in diploid hybrids and the presence of a suppressor system promoting bivalent pairing in allopolyploids.

When Poggio *et al.* (1993) studied the meiotic behaviour of several natural diploid F1 *G. pulchella* × *G. incisa*, marked differences were observed in the frequency of bivalents, univalents and quiasmata. These hybrids varied from complete to almost no homoeologous chromosome pairing. To explain these results, Poggio *et al.* (1993), suggested that some genetic combinations of parental species are more disharmonious than others and postulated that parental species could be polymorphic and/or polytypic for mutations that cause changes in the site of attachment of chromosomes to the nuclear membrane. Variability in chromosome pairing in F1 hybrids has also been noted in other plants too (Jahuar and Joppa, 1996).

In this work, the meiotic behaviour of natural and artificial hybrids between the diploid species *G. incisa* and *G. pulchella* is reported. The aim is to clarify mechanisms involved in the variability of the meiotic chromosome pairing amongst F1 hybrids. The presence and activity of pairing regulator gene/s at diploid level are discussed in the light of these findings.

## Methods

All plant material was collected in the Capital Department of the Province of Corrientes, Argentina. *G. incisa*, *G. pulchella* and natural hybrids H1–H6 were collected in 1991 and new individuals of *G. incisa*, *G. pulchella* and natural hybrids H7, H8 and H9 in 2011. The minimum distance between collected samples was 20  m. Some individuals of *G. incisa*, *G. pulchella* and hybrids collected in Corrientes were subsequently cultivated at the ‘Instituto Fitotécnico de Santa Catalina’ Llavallol, Province of Buenos Aires, Argentina.

Distance between Corrientes collection and the ‘Instituto Fitotécnico de Santa Catalina’, is ∼1100 Km and the two localities belong to different Phytogeographic Regions (Cabrera 1994). Herbarium material was deposited in Facultad de Ciencias Exactas y Naturales, Universidad de Buenos Aires, Ciudad Autónoma de Buenos Aires, Argentina.Taxonomic identification of the species and natural and artificial hybrids was made according morphological criteria in Poggio *et al.* (1993). No evidence of later generation of progeny or introgression was detected.

### Artificial hybrids

*G. pulchella* was used as mother because its pistil is shorter than in *G. incisa* and it was reported previously that crosses are successful when the mother is the species with the shortest pistil (Schnack and Covas 1945a,b). Styles of *G. pulchella* were pollinated repeatedly for about 7 days using *G. incisa* as pollen donor. Most crosses were unsuccessful but three different parental pairings produced fruit. One of these crosses produced seven sibling hybrids (H10–H16). In the other two crosses only one hybrid from each was obtained (H17 and H18). Parental and hybrids of the three artificial crosses were cultivated in different greenhouses, isolated from each other.

### Cytological analysis

For meiotic studies, immature flowers were fixed in absolute ethanol: acetic acid (3:1 vol/vol) and the anthers squashed in 2% acetic haematoxylin. Slides were made permanent by freezing with liquid CO_2_, removing the coverslip, dehydrating in absolute alcohol and mounting in Euparal.

### Statistical analysis

The differences between species in the number of bivalents, univalents, closed bivalents and quiasmata per cell were tested with analyses of variance. The means of cytogenetic parameters were calculated and multiple contrasts were performed using the LSD Fisher method (Fisher 1932). These statistical analyses were considered significant if *P* values were < 0.05. Methods of Sneath and Sokal (1973) were used to estimate similarities between hybrids according to chromosome behaviour in metaphase I. Data Basic Matrix (*n* × *t* matrix) was constructed, their *t* columns represented the 12 cytogenetical parameters (mean value/cell and maximum and minimum values of bivalents, closed bivalents, univalents and chiasmata) and *n* rows were the 18 operational taxonomic units (OTU’s) constituted by the hybrids. The Similarity Coefficients used were Manhattan, Gower and Euclidean. The resulting OTU × OTU matrix served as input in the calculation of dendrogram by the unweighted pair-group method, using arithmetic averages. The Cophenetic Correlation Coefficient (*r*) was computed as a measurement of distortion for each one of the methods used. The statistical analyses were performed using the Infostat programme, FCA, National University of Córdoba (Di Rienzo *et al.* 2015).

## Results

**Figure 1.**
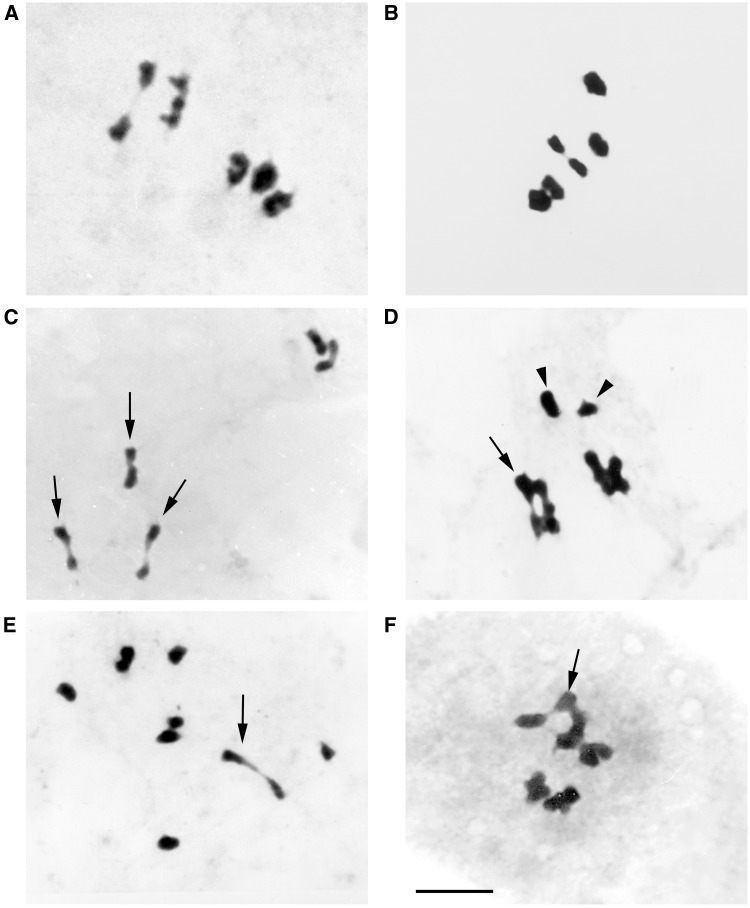
Meiotic behavior in *G. incisa, G. pulchella* and their hybrids. (**A)** *G. incisa* (5II); (**B)** *G. pulchella* (5II); (**C)** H8 (5II); (**D)** H7 (4II + 2I); (**E)** H18 (2II + 6I); (**F)** H16 (5II). The arrows show the most conspicuous heteromorphic bivalents., Arrow heads show two homoeologous heteromorphic univalents. The bar represents 10 µm. All photographs with the same enlargement.

Figure 1 shows metaphase I cells of *G. incisa*, *G. pulchella* and their natural and artificial hybrids. Parental species have five bivalents (Fig. 1A and B) and the hybrids have a variable number of bivalents (Fig. 1C–F). In the hybrids, most of the bivalents are heteromorphic. Table 1 presents the chromosome behavior in metaphase I of the parental species and their hybrids. Cytogenetic parameters show significant differences among taxa (bivalents per cell, *F*_21,1477__ _= 373, *P* < 0.0001; univalents per cell *F*_21,1477_ = 386.49, *P* 0.0001; closed bivalent per cell *F*_21,1477 _ = 62.12, *P* < 0.0001; chiasmata per cell *F*_21,1477_ = 155.75, *P* < 0.0001). The contrasts performed with the LSD Fisher method are presented in Table 1.

**Table 1.** Chromosome behavior in *G. incisa, G. pulchella* and natural and artificial hybrids.

Species and hybrids	Meiotic configuration at Metaphase I			Quiasmata/cell mean ± SD (range)	No of plants (No of cells)
	II/cell	I/cell	IIc/cell		
	Mean ± SD (range)	Mean ± SD (range)	Mean ± SD (range)		
*G. incisa*^a^	5.00 ± 0.00^G^	0.00 ± 0.00^A^	2.75 ± 0.86^J^ (0–5)	7.75 ± 0.86^I^ (5–10)	8 (338)
G. pulchella*	5.00 ± 0.00^G^	0.00 ± 0.00^A^	1.57 ± 0.96^D^ (0–5)	6.57 ± 0.96^F^ (5–10)	10 (382)
H1^a^	1.08 ± 0.49^A^ (0–3)	7.84 ± 0.99^G^ (4–10)	0.00 ± 0.00^A^	1.08 ± 0.49^A^ (0–3)	1 (37)
H2^a^	4.06 ± 0.80^F^ (2–5)	1.89 ± 1.60^B^ (0–6)	1.33 ± 1.19^BCD^ (0–5)	5.39 ± 1,50^D^ (4–10)	1 (18)
H3^a^	3.29 ± 1.15^D^ (1–5)	3.43 ± 2.29^D^ (0–8)	0.86 ± 1.11^B^ (0–3)	4.14 ± 1.53^C^ (1–8)	1 (21)
H4^a^	4.93 ± 0.26^G^ (4–5)	0.14 ± 0.51^A^ (0–2)	3.10 ± 0.75^I^ (1–5)	8.03 ± 0.78^J^ (6–10)	1 (100)
H5^a^	4.98 ± 0.16^G^ (4–5)	0.05 ± 0.31^A^ (0–2)	1.04 ± 1.02^BC^ (0–4)	6.01 ± 1,04^E^ (5–9)	1 (82)
H6^a^	4.86 ± 0.35^G^ (4–5)	0.28 ± 0.65^A^ (0–2)	1.34 ± 0.86^BCD^ (0–3)	6.21 ± 0,98^EF^ (4–8)	1 (29)
H7^b^	3.50 ± 0.72^E^ (2–5)	3.00 ± 0,0.72^C^ (0–6)	0.00 ± 0.00^A^	3.50 ± 0.72^B^ (2–5)	1 (24)
H8^b^	5.00 ±0.00^G^	0.00 ± 0.00^A^	1.28 ± 1.27^BCD^ (0–5)	6.28 ± 1.27^EF^ (5–10)	1 (18)
H9^b^	2.80 ± 1.21^B^ (1–5)	4.40 ± 2.41^F^ (0–8)	1.60 ± 0.83^DE^ (1–4)	4.40 ± 1.55^C^ (3–8)	1 (15)
*G. incisa*^c^	5.00 ± 0.00^G^	0.00 ± 0.00^A^	4.14 ± 0.64^H^ (3–5)	9.14 ± 0.64^K^ (8–10)	1 (29)
*G. pulchella*^c^	5.00 ± 0.00^G^	0.00 ± 0.00^A^	1.58 ± 1.06^D^ (0–4)	6.58 ± 1.06^F^ (5–9)	1 (31)
H10^c^	5.00 ± 0.00^G^	0.00 ± 0.00^A^	2.25 ± 1.02^FG^ (0–4)	7.25 ± 1.02^GH^ (5–9)	1 (32)
H11^c^	5.00 ± 0.00^G^	0.00 ± 0.00^A^	2.59 ± 0.68^GH^ (2–4)	7.59 ± 0.68^GHI^ (7–9)	1 (29)
H12^c^	5.00 ± 0.00^G^	0.00 ± 0.00^A^	3.04 ± 0.75^I^ (2–5)	8.04 ± 0.75^J^ (7–10)	1 (71)
H13^c^	5.00 ± 0.00^G^	0.00 ± 0.00^A^	2.65 ± 1.14^GH^ (0–5)	7.65 ± 1.14^HI^ (5–10)	1 (40)
H14^c^	5.00 ± 0.00^G^	0.00 ± 0.00^A^	2.26 ± 0.98^FG^ (1–4)	7.26 ± 0.98^GH^ (6–9)	1 (53)
H15^c^	5.00 ± 0.00^G^	0.00 ± 0.00^A^	3.00 ± 1.10^HI^ (1–5)	8.00 ± 1.10^IJ^ (6–10)	1 (31)
H16^c^	5.00 ± 0.00^G^	0.00 ± 0.00^A^	2.13 ± 1.00^EF^ (0–4)	7.13 ± 1.00^G^ (5–9)	1 (39)
H17^c^	2.95 ± 0.92^BC^ (1–5)	4.10 ± 1.84^EF^ (0–8)	1.46 ± 0.78^D^ (0–4)	4.41 ± 1.22^C^ (2–8)	1 (41)
H18^c^	3.08 ± 1.22^C^ (1–5)	3.85 ± 2.44^E^ (0–8)	1.36 ± 0,67^CD^ (1–4)	4.44 ± 1.50^C^ (2–8)	1 (39)

^a^Data from Poggio et al. (1993).

^b^Natural hybrids collected in Province of Corrientes, Argentina.

^c^Artificial hybrids and their parental species cultivated in Instituto Fitotécnico de Santa Catalina, Prov. of Buenos Aires, Argentina. II, bivalents; I, univalents; II_c _=_ _closed bivalents. Per column, means with the same letter are not significantly different (*P* ≤ 0.05).

The three new natural hybrids collected in the same area and several years later than those collected by Poggio *et al.* (1993) present significant differences in the frequency of bivalents, univalents, closed bivalents and quiasmata (Table1). Two of these hybrids (H7 and H9) have irregular meiosis with a high number of univalents in metaphase I, while the remaining (H8) shows five bivalents in 100% of the metaphase I (Table1). Nine artificial hybrids were obtained from three crosses. Two of the hybrids (H17 and H18) came from two different crosses and differ significantly in the frequency of bivalents, univalents, closed bivalents and quiasmata from the remaining seven hybrids (sibling, H10–H16) (Table 1). These sibling hybrids came from only one cross and show very similar meiotic behaviour.

Parental species, the natural hybrid H8 and the seven sibling hybrids (H10–H16) show five bivalents in 100% of their metaphase I. The rest of natural and artificial hybrids give a frequency of bivalents per cell that range from 1.08 to 4.98. Multivalent configurations were not observed in the 780 parental cells or in the 719 hybrid cells.

**Figure 2.**
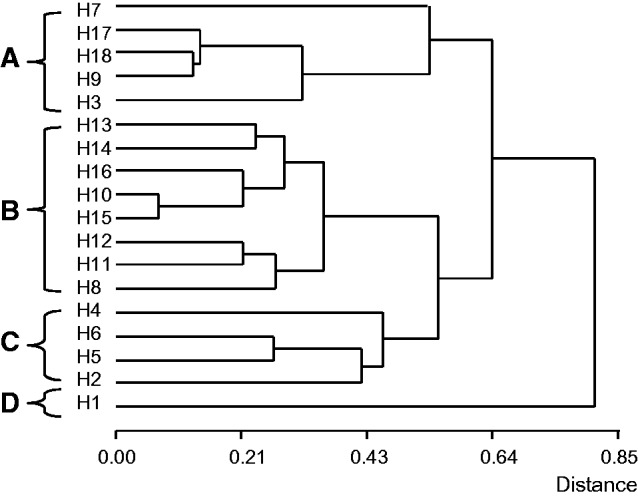
Dendrogram showing the similarity of hybrids according to the meiotic behavior of their chromosomes. **(A)** Hybrids with a range of bivalents/cell between 2.85 and 3.50. **(B)** Hybrids with five bivalents/cell in all their metaphase I. **(C)** Hybrids with a range of bivalents/cell between 4.05 and 4.93. **(D)** Hybrid with the minor bivalent percentage value. Cophenetic Correlation Coefficient *r* = 0.925.

Dendrograms constructed using a Data Basic Matrix of 12 cytogenetical parameters, eighteen OTUs (hybrids) and three Similarity Coefficients generate the same general structure. Figure 2 shows the dendrogram obtained applying the Gower coefficient, which has the higher Cophenetic Correlation Coefficient of 0.92. Four main groups are observed, A–D. Each of these groups is formed by hybrids with a high level of similarity according to their chromosome behaviour in metaphase I. The group A (H7, H17, H18, H9, H3), comprises hybrids showing a range of bivalents between 2.85 and 3.50. The group B (H13, H14, H16, H10, H15, H12, H11, H8) is formed by hybrids with only five bivalents. The group C (H4, H6, H5, H2) is composed by hybrids with a range of bivalent between 4.05 and 4.98. The group D has only one hybrid (H1) which has the minor value of the percentage of bivalents (Table 1). The highest distance is between H1 and all the other hybrids.

## Discussion

The analysis of the meiotic behaviour of species, hybrids and polyploids of *Glandularia* made by different authors generated questions about the mechanisms involved in homologous/homoeologous chromosome pairing (Poggio *et al.* 1993; Schnack and Covas 1945a,b; Schnack and González 1945; Schnack and Solbrig 1953; Schnack *et al.* 1959; Solbrig *et al.* 1968). The homoeologous pairing in interspecific diploid hybrids and the diploidized meiotic behaviour described in polyploid species lead us to propose that *Glandularia* species have a Ph-like gene, regulating homologous/homoelogous pairing in a similar way than that described for wheat. Genetic determination of exclusive pairing between homologous chromosomes was described in several species of polyploid origin (*Triticum aestivum*, *Triticum turgidum*, *Festuca arundinacea*, *Avena sativa*, *Brassica napus*, *Gossypium hirsutum* and *Glandularia* *barbadense*) (Bhullar *et al.* 2014; Cifuentes *et al.* 2010; Jenczewski *et al.* 2003; Jenczewski and Alix 2004). However little is known about mechanisms of pairing when homoeologous genomes are in only one dose, i.e. in diploid nuclei. In *Glandularia, s*everal artificial interspecific diploid hybrids, described by different authors, displayed homoeologous pairing, with the formation of five bivalents in most of the cells. Interestingly, unexpected differences in the frequency of bivalents, univalents and quiasmata were reported by Poggio *et al.* (1993) in natural diploid hybrids between *G. pulchella* and *G. incisa.* The parental species and hybrids reported by these authors grew in sympatry in a small geographical area and they suggested that some genetic combinations of parental species were more disharmonious than others and postulated that parental species would be polymorphic and/or polytypic for mutations affecting chromosome pairing.

The meiotic behavior of new natural and artificial F1 hybrids *G. pulchella* × *G. incisa* allowed us to explore in greater depth the nature of the variability in the chromosome pairing in diploid hybrids. Significant differences in the frequency of bivalents, univalents, closed bivalents and quiasmata were detected in the three new natural hybrids collected in the same area and several years later than those collected by Poggio *et al.* (1993). Moreover artificial hybrids were performed with the aim to explore different genotypic combinations. Variability in the frequency of bivalents, univalent, closed bivalents and quiasmata were observed in hybrids coming from different crosses. Conversely, sibling hybrids which came from only one cross presented a meiotic behaviour similar between them.

These results generate questions about the causes of variability in the amount of homoeologous pairing in natural and artificial diploid hybrids. The amount of chromosome pairing seemingly depends not just on the degree of chromosome homology. Chromosome size or genome size differences between parental species, as well as cytoplasmic effects and environmental factors influence the amount of chromosome pairing and chiasmata frequency.

Parental species, *G incisa* and *G pulchella*, differ significantly in 2C DNA content (Poggio *et al.* 1992). This difference in the genome size was not a critical factor for pairing failure, being heteromorphic most of the bivalents in the hybrids. Moreover, it can be assumed that all the hybrids possessed the same degree of structural homoeology, since cytological evidence for structural re-arrangements was never found in the parental species. Environmental factors would not explain the variability observed in the meiotic behavior of *Glandularia* hybrids we studied since they grew in sympatry in a restricted area that had homogeneous ecological conditions.

Given these considerations, we suggest that the make-up of the parental genome influences the activity of pairing regulator genes and is thus mainly responsible for the variability in the amount of homoeologous pairing in the hybrids. This conclusion is strongly supported by the meiotic analysis of the seven artificial sibling hybrids. These had the same genotypical combination and showed similar meiotic variability between them, confirming that genotype combination of the parents is the key to understand variability in the chromosome pairing of the F1 hybrids.

The different meiotic phenotypes of the hybrids were grouped according to their chromosome behaviour in metaphase I. The dendrogram obtained using means per cell and maximum and minimum values of cytological parameters (bivalents, closed bivalents, univalents and chiasmata) showed four main clusters that revealed different meiotic phenotypes. Interestingly, each cluster was formed by hybrids with a high level of similarity mainly in their mean number of paired chromosomes in metaphase I. The high Cophenetic Correlation Coefficient obtained (0.92) allow us to infer that the dendrogram is an accurate representation of the true meiotic similarities between hybrids.

Different meiotic phenotypes were also observed in *B.* *napus*. The allotetraploid (2*n* = 38, AACC) shows complete diploid-like meiotic behaviour and produced haploids (AC) (*n* = 19) with two main phenotypes depending on the variety they were isolated. Some haploids showed 8.1-13.8 univalents at metaphase while others showed only 2.4–5.7 univalents. This demonstrates that A and C genomes are homoeologous and prone to recombine and that the differences in the meiotic behaviour at metaphase I in different haploids reflect differences in pairing between homoeologous chromosomes. On basis of these results, a pairing regulator locus with incomplete penetrance and an effect dosage sensitive was proposed for *B.* *napus* (reviewed by Cifuentes *et al.* 2010; Jenczewski *et al.* 2003).

The pairing regulator gene/s that we propose for *Glandularia* resembles that of the *B. napus* since both preclude homoeologous pairing when homologous genomes are in two doses in the polyploids and both display incomplete penetrance when homologous genomes are in one dose in the diploids.

The mechanisms controlling genes that regulate pairing are still unclear and several models have been proposed for the mode of action of Ph1 of wheat Feldman and Levy (2012) published a model where Ph1 affects the pre-meiotic alignment of homologous and homoeologous chromosomes showing that different doses of Ph1 affect the distance between them. In *Glandularia* Poggio *et al.* (1993) proposed that genes affecting chromosome distribution in the nuclear membrane could modify the distance between homologous and/or homoeologous genomes. Recently we found evidence for spatial separation of haploid groups of five chromosomes in the hybrids (in preparation), a phenomena well documented in other plant groups (Callimassia *et al.* 1994; Ding *et al.* 2014; Ran *et al.* 2001). The separation of parental chromosomes potentially restricts homoeologous pairing and favors homologous pairing, resulting in balanced chromosome segregation in allopolyploids. On this basis we propose that pairing in *Glandularia* diploid hybrids depends on the distance between nuclear site attachment of its two parental genomes and that this distance would depend of the genetic make-up constitution of parental species which would influence the activity of pairing regulator genes.

The molecular and cell biological characterization of Ph1 of wheat lead Cifuentes *et al.* (2010) to infer its involvement in fundamental mechanisms which could be conserved across kingdoms. In relation to the origin of Ph-like regulators in polyploids, several authors suggested that these loci may already have been present in some diploid progenitor genotypes, while others proposed that they developed after the origin of the polyploidy species (reviewed in Jenczewski and Alix, 2004).

We suggest that Ph-like regulators present in *Glandularia* originated in diploid South American species. This proposal is based on morphological, molecular and cytogenetical evidence indicating that the genus *Glandularia* originated on the South American continent where the species are largely diploid (Lewis and Oliver 1961; Solbrig *et al.* 1968; Umber 1979; Yuan and Olmstead 2008). Furthermore, morphological and cytogenetical data obtained by Solbrig *et al.* (1968) demonstrated that the diploid *G. pulchella*, studied in the present work, was an ancestor of *G. elegans*, a North American hexaploid species that showed a completed diploid-like meiotic behaviour.

## Conclusions

Analysis of the meiotic behaviour of species, natural and artificial hybrids and polyploids of *Glandularia* strongly suggests the activity of pairing regulator genes (Ph-like) prevents homoeologous pairing when homologous genomes are in two doses in polyploids. In this work, we postulate, in diploid species, the presence of pairing regulator genes which possess an incomplete penetrance when homologous genomes are presence only once. Moreover, we postulate that influences on the activity of pairing regulator genes determined by the genetic make-up of parental species are mainly responsible for differences in the amount of homoeologous pairing between diploid hybrids. Based on phylogenetic, cytological and morphological studies we suggest that the Ph-like genes of *Glandularia* originated in South American diploid species. The results obtained in this work, together with the small number of chromosomes and the relative ease with which artificial hybrids and polyploids can be generated in *Glandularia* suggest this genus is a good model to analyse and explore the activity of pairing regulator genes at different ploidy levels.

## Sources of Funding

Funding was provided by grants from the Consejo Nacional de Investigaciones Científicas y Técnicas (CONICETPIP 00342), Universidad de Buenos Aires (UBACYT 20020100100859) and Agencia Nacional de Producción Científica y Tecnológica—SECyT (PICT 2010-1665).

## Conflict of Interest Statement

None declared.

## References

[plw030-B1] Arora OP. 1978 Cytogenetic analysis of *Verbena teasii.* Cytologia 43:91–96.

[plw030-B2] Bhullar R Nagarajan R Bennypaul H Sidhu GK Sidhu G Rustgi S Gill KS. 2014 Silencing of a metaphase I-specific gene results in a phenotype similar to that of the Pairing homeologous 1 (Ph1) gene mutations. Proceedings of the National Academy of Sciences of United States of America 111:14187–14192. 10.1073/pnas.1416241111 PMC4191769 25232038

[plw030-B3] Cabrera AL. 1994 Regiones fitogeográficas argentinas In Kugler WF, ed. Enciclopedia argentina de agricultura y jardinería. Acme. Buenos Aires. Argentina: Fascículo 1, 1–85.

[plw030-B4] Callimassia MA Murray BG Hammett KRW Bennett MD. 1994 Parental genome separation and asynchronous centromere division in interspecific F1 hybrids in *Lathyrus.* Chromosome Research 2:383–397. 7981943 10.1007/BF01552798

[plw030-B5] Cifuentes M Grandont L Moore G Chèvre AM Jenczewski E. 2010 Genetic regulation of meiosis in polyploid species: new insights into an old question. New Phytologist 186:29–36. 19912546 10.1111/j.1469-8137.2009.03084.x

[plw030-B6] Colas I Shaw P Prieto P Wanous M Spielmeyer W Mago R Moore G. 2008 Effective chromosome pairing requires chromatin remodeling at the onset of meiosis. Proceedings of the National Academy of Sciences United States of America 105:6075–6080. 10.1073/pnas.0801521105 PMC2329686 18417451

[plw030-B7] Ding L Zhao ZG Ge XH Li ZY. 2014 Different timing and spatial separation of parental chromosomes in intergeneric somatic hybrids between *Brassica napus* and *Orychophragmus violaceus.* Genetics and Molecular Research 13:1618–2611. 10.4238/2014.April.8.3 24782049

[plw030-B8] Di Rienzo JA Casanoves F Balzarini MG Gonzalez L Tablada M Robledo CW. InfoStat versión 2015 Argentina: Grupo InfoStat, FCA, Universidad Nacional de Córdoba. URL http://www.infostat.com.ar.

[plw030-B9] Feldman M Levy AA. 2012 Genome evolution due to allopolyploidization in wheat. Genetics 192:763–774. 23135324 10.1534/genetics.112.146316 PMC3522158

[plw030-B10] Fisher RA. 1932 Statistical methods for research workers. 4th edn Edinburgh: Oliver and Boyd.

[plw030-B11] González Roca L Iannicelli J Coviella A Bugallo V Bologna P Pitta-Álvarez S Escandón A. 2015 A protocol for the *in vitro* propagation and polyploidization of an interspecific hybrid of *Glandularia* (*G. peruviana* x *G. scrobiculata*). Scientia Horticulturae 184:46–54.

[plw030-B12] Holm PB Wang X. 1988 The effect of chromosome 5B on synapsis and chiasma formation in wheat, *Triticum aestivum cv. Chinese Spring.* Carlsberg Research Communications 53:191–208.

[plw030-B13] Imhof L Suarez M Paganelli F Facciuto G. 2013 Interspecific hybridization among three species of *Glandularia* (Verbenaceae) native to Argentina. Acta Horticulturae 1000:481–486.

[plw030-B14] Jauhar PP Joppa LR. 1996 Chromosome pairing as a tool in genome analysis: Merits and Limitations In Jauhar PP, ed. Methods of genome analysis in plants. New York: CRC Press, Inc, 9–39.

[plw030-B15] Jenczewski E Eber F Grimaud A Huet S Lucas MO Monod H Chevre AM. 2003 PrBn, a major gene controlling homeologous pairing in oilseed rape (*Brassica napus*) haploids. Genetics 164:645–653. 12807785 10.1093/genetics/164.2.645 PMC1462591

[plw030-B16] Jenczewski E Alix K. 2004 From diploids to allopolyploids: the emergence of efficient pairing control genes in plants. Critical Reviews in Plant Sciences 23:21–45.

[plw030-B17] Khoshoo TN Arora OP. 1969 Genesis of bivalent pairing in hexaploid clump *Verbena.* Chromosoma 26:259–269.

[plw030-B18] Lewis WH Oliver RL. 1961 Cytogeography and Phylogeny of the North American Species of *Verbena.* American Journal of Botany 48:638–643.

[plw030-B19] Mikhailova EI Naranjo T Shepherd K Wennekes-van Eden J Heyting C De Jong JH. 1998 The effect of the wheat Ph1 locus on chromatin organisation and meiotic chromosome pairing analysed by genome painting. Chromosoma 107:339–350. 9880767 10.1007/s004120050316

[plw030-B20] O’Leary N Peralta P. 2007 Nuevas combinaciones en el género *Glandularia* (Verbenaceace). Darwiniana 45:218–230.

[plw030-B21] Peralta PF Múlgura ME. 2011 El Género *Glandularia* (Verbenaceae) en Argentina. Annals of the Missouri Botanical Garden 98:358–412.

[plw030-B22] Poggio L Greizerstein RJ Ferrari MR. 1992 Meiotic variability in diploids F1 hybrids in *Glandularia* (Verbenaceae). 11th. International Chromosome Conference. Edinburg, Scotland, U.K. Abstract:32.

[plw030-B23] Poggio L Botta SM Greizerstein EJ Ferrari MR. 1993 Natural hybridization in *Glandularia* (Verbenaceae). Evolutionary implications of chromosome pairing. Darwiniana 32:77–90.

[plw030-B24] Ran Y Hammett KR Murray BG. 2001 Hybrid identification in *Clivia* (Amaryllidaceae) using chromosome banding and genomic *in situ* hybridization. Annals of Botany 87:457–462.

[plw030-B25] Schnack B Covas G. 1945a Hibridación interespecífica en *Glandularia* (Verbenaceas*).* Darwiniana 7:71–79.

[plw030-B26] Schnack B Covas G. 1945b Un híbrido interespecífico del género *Glandularia* *Revista. Argentina de Agronomía. Universidad Nacional de La Plata* 12**:** 224–229.

[plw030-B27] Schnack B González FF. 1945 Estudio morfológico y citogenético del híbrido Glandularia santiaguensis x G. megapotamica. Revista. Argentina de Agronomía. Universidad Nacional de La Plata 12:285–291.

[plw030-B28] Schnack B Solbrig OT. 1953 El híbrido Glandularia laciniata x G. peruviana y su anfidiploide artificial. Revista de la Facultad de Agronomía. Universidad Nacional de La Plata 29:255–266.

[plw030-B29] Schnack B Fehleisen S. 1955 Observaciones en poliploides del género Glandularia (Verbenaceae). *Revista de la Facultad de Agronomía* *Universidad Nacional de La Plata* 31:39–52.

[plw030-B30] Schnack B Fehleisen S Cocucci AE. 1959 Estudios del híbrido interespecífico *Glandularia canadensis* (L.) Small. x *G. peruviana* (L.) Small. *Revista de la Facultad de Agronomía. Universidad Nacional de La Plata* 35:113–121.

[plw030-B31] Sneath PHA Sokal RR. 1973 Numerical taxonomy: the principles and practice of numerical classification, 1st edn San Francisco: WH. Freeman and Company.

[plw030-B32] Solbrig OT Passani C Glass R. 1968 Artificial hybridization between different polyploid levels in *Glandularia* (Verbenaceae). American Journal of Botany 55:1235–1239.

[plw030-B33] Turner BL Powell AM. 2005 Chromosome numbers of *Glandularia* (Verbenaceae) from Central and trans-Pecos, Texas. *SIDA.* Contributions to Botany 21:1657–1661.

[plw030-B34] Umber RE. 1979 The genus *Glandularia* (Verbenaceae) in North America. Systematic Botany 4:72–102.

[plw030-B35] Yuan Y Olmstead RG. 2008 A species-level phylogenetic study of the *Verbena* complex (Verbenaceae) indicates two independent intergeneric chloroplast transfers. Molecular Phylogenetics and Evolution 48:23–33. 18495498 10.1016/j.ympev.2008.04.004

[plw030-B36] Zhou A Pawlowski WP. 2014 Regulation of meiotic gene expression in plants. Frontiers in Plant Science 5:413. 25202317 10.3389/fpls.2014.00413 PMC4142721

